# *C*-class functions with new approach on coincidence point results for generalized $(\psi ,\varphi )$-weakly contractions in ordered *b*-metric spaces

**DOI:** 10.1186/s40064-016-2481-1

**Published:** 2016-06-21

**Authors:** Zead Mustafa, Mohammed M. M. Jaradat, Arslan Hojat Ansari, Branislav Z. Popović, Husein M. Jaradat

**Affiliations:** Department of Mathematics, Statistics and Physics, Qatar University, Doha, Qatar; Department of Mathematics, Karaj Branch, Islamic Azad University, Karaj, Iran; Department of Mathematics and Informatics, University of Kragujevac, Radoja Domanovića 12, Kragujevac, 34 000 Serbia; Department of Mathematics, Al al-Bayt University, Mafraq, Jordan; Department of Mathematics and Applied Sciences, Dhofar University, Salalah, Oman

**Keywords:** *b*-Metric space, Partially ordered set, Fixed point, *C*-Class functions, Primary 47H10, Secondary 54H25

## Abstract

In this paper, by using the *C*-class functions and a new approach we present some coincidence point results for four mappings satisfying generalized $(\psi ,\phi )$-weakly contractive condition in the setting of ordered b-metric spaces. Also, an application and example are given to support our results.

## Background

Metric fixed point theorem is playing a major role in mathematics and the applied sciences. Over the past two decades the development of fixed point theory in metric spaces has attracted considerable attention due to many applications in different areas such as variational, linear inequalities and optimization problems.

Banach contraction principle states that every contractive mapping defined on a complete metric space has a unique fixed point. This principle has been generalized by many researchers in different ways Abbas and Dorić (2010), Abbas et al. (2011), Abbas et al. (2012), Abbas and Rhoades (2009), Agarwal et al. (2008) and Shatanawi and Postolache (2013), Shatanawi et al. (2011), Shatanawi and Mustafa (2012), Choudhury et al. (2013), Aydi et al. (2013), Aydi et al. (2012), Shatanawi et al. (2014), Radenović and Kadelburg (2012).

In 1997, Alber and Guerre-Delabriere (1997) introduced the concept of weak contraction in the setup of Hilbert spaces as follows: A self mapping *f* on *X* is a weak contraction, if $d(fx,fy)\le d(x,y)-\varphi (d(x,y))$ for all $x,y\in X$, where $\varphi$ is an altering distance function. Thereafter, in Rhoades (2001), generalized the Banach contraction principle by considering the class of weak contraction in the setup of metric spaces and proved that every weakly contractive mapping defined on a complete metric space has a unique fixed point.

Later on, in Zhang and Song (2009) introduced the concept of a generalized $\varphi$-weak contractive mappings and proved the following common fixed point result: Let (*X*, *d*) be a complete metric space. If $f,\,g:X\rightarrow X$ are generalized $\varphi$-weak contractive mappings, then there exists a unique point $u\in X$ such that $u=fu=gu.$

We refer the reader to Abbas and Dorić (2010), Dorić (2009), Moradi et al. (2011) and Razani et al. (2012) for more works in this area.

The concept of *b*-metric space was introduced by Czerwik in Czerwik (1998). Since then, several papers have been published on the fixed point theory of various classes of single-valued and multi-valued operators in *b*-metric spaces (see also Akkouchi 2011; Aydi et al. 2012; Boriceanu 2009a, b; Boriceanu et al. 2010; Bota et al. 2011; Hussain et al. 2012; Hussain and Shah 2011; Olatinwo 2008; Mustafa 2014; Pacurar 2010; Mustafa 2013; Ansari et al. 2014).

## Mathematical preliminaries

### **Definition 1**

(Altun and Simsek 2010) Let *f* and *g* be two selfmaps on partially ordered set *X*. A pair (*f*, *g*) is said to be weakly increasing if $fx\preceq gfx$ and $gx\preceq fgx$ for all $x\in X$.

### **Definition 2**

(Abbas et al. 2011) Let *f* and *g* be two selfmaps on partially ordered set *X*. A pair (*f*, *g*) is said to be partially weakly increasing if $fx\preceq gfx$ for all $x\in X$.

Let *X* be a non-empty set and $T:X\rightarrow X$ be a given mapping. For every $x\in X$, let $T^{-1}(x)=\{u\in X:Tu=x\}$.

### **Definition 3**

(Nashine and Samet 2011) Let $(X,\preceq )$ be a partially ordered set and $f,g,T:X\rightarrow X$ are mappings such that $fX\subseteq TX$ and $gX\subseteq TX$. The ordered pair (*f*, *g*) is said to be weakly increasing with respect to *T* if and only if for all $x\in X$, $fx\preceq gy$ for all $y\in T^{-1}(fx)$ and $gx\preceq fy$ for all $y\in T^{-1}(gx)$.

### **Definition 4**

(Esmaily et al. 2012) Let $(X,\preceq )$ be a partially ordered set and $f,g,T:X\rightarrow X$ are mappings such that $fX\subseteq TX$ and $gX\subseteq TX$. The ordered pair (*f*, *g*) is said to be partially weakly increasing with respect to *T* if $fx\preceq gy$ for all $y\in T^{-1}(fx)$.

### *Remark 5*

In the above definitions:

1. If $f=g$, we say that *f* is weakly increasing (partially weakly increasing) with respect to *T*.
2. If $T=I_X$ (the identity mapping on *X*), then the above definitions reduces to the weakly increasing (partially weakly increasing) mapping (See, Nashine and Samet 2011; Shatanawi and Samet 2011).

Jungck in Jungck (1986) introduced the following definition.

### **Definition 6**

(Jungck 1986) Let (*X*, *d*) be a metric space and $f, g:X\rightarrow X$. The pair (*f*, *g*) is said to be compatible if $\lim \nolimits _{n\rightarrow \infty }d(fgx_n,gfx_n)=0$, whenever $\{x_n\}$ is a sequence in *X* such that $\lim \nolimits _{n\rightarrow \infty }fx_n=\lim \nolimits _{n\rightarrow \infty }gx_n=t$ for some $t\in X$.

### **Definition 7**

Let *f* and *g* be two self mappings on a nonempty set *X*. If $x=fx=gx$ for some *x* in *X*, then *x* is called a common fixed point of *f* and *g*.

### **Definition 8**

(Jungck 1996) Let $f,g:X\rightarrow X$ be given self-mappings on *X*. The pair (*f*, *g*) is said to be weakly compatible if *f* and *g* commute at their coincidence points (i.e., $fgx=gfx$, whenever $fx=gx$).

### **Definition 9**

Let $(X,\preceq )$ be a partially ordered set and *d* be a metric on *X*. We say that $(X,d,\preceq )$ is regular if the following conditions hold:

1. If a non-decreasing sequence $x_n\rightarrow x$, then $x_n\preceq x$ for all *n*.
2. If a non-increasing sequence $y_n\rightarrow y$, then $y_n\succeq y$ for all *n*.

### **Definition 10**

(Khan et al. 1984) A function $\psi : [0,\infty ) \rightarrow [0,\infty )$ is called an altering distance function if it satisfies the following conditions:

1. $\psi$ is monotone increasing and continuous,
2. $\psi (t) =0$ if and only if $t = 0$.

In Nashine and Samet (2011), established some coincidence point and common fixed point theorems for mappings satisfying a generalized weakly contractive condition in an ordered complete metric space by considering a pair of altering distance functions $(\psi ,\varphi )$. In fact, they proved the following theorem.

### **Theorem 11**

(Nashine and Samet 2011 Theorem 2.4.) *Let*$(X,\preceq )$*be a partially ordered set and suppose that there exists a metricdonXsuch that* (*X*, *d*) *is a complete metric space. Let*$T,R:X\rightarrow X$*be given mappings satisfying for every pair*$(x,y)\in X\times X$*such thatRxandRyare comparable*,

$$\begin{aligned} \psi (d(Tx,Ty))\le \psi (d(Rx,Ry))-\varphi (d(Rx,Ry)), \end{aligned}$$

*where*$\psi$*and*$\varphi$*are altering distance functions. We suppose the following hypotheses:*

(i) *TandRare continuous*,
(ii) $$TX\subseteq RX$$,
(iii) *Tis weakly increasing with respect toR*,
(iv) *the pair* (*T*, *R*) *is compatible.*

*Then*, *TandRhave a coincidence point, that is, there exists*$u\in X$*such that*$Ru=Tu.$

Further, they showed that by replacing the continuity hypotheses on *T* and *R* with the regularity of $(X,d,\preceq )$ and omitting the compatibility of the pair (*T*, *R*), the above theorem is still valid (see, Theorem 2.6 of Nashine and Samet 2011).

Also, in Shatanawi and Samet (2011), Shatanawi and Samet studied common fixed point and coincidence point for three self mappings *T*, *S* and *R* satisfying $(\psi , \varphi )$-weakly contractive condition in an ordered metric space (*X*, *d*), where *S* and *T* are weakly increasing with respect to *R* and $\psi ,\varphi$ are altering distance functions. Their result generalize Theorem 11.

Analogous to the work in Nashine and Samet (2011), Shatanawi and Samet proved the above result by replacing the continuity hypotheses of *T*, *S* and *R* with the regularity of *X* and omitting the compatibility of the pair (*T*, *R*) and (*S*, *R*) (See, Theorem 2.2 of Shatanawi and Samet 2011).

Consistent with Czerwik (1998), Jovanović et al. (2010) and Singh and Prasad (2008), the following definitions and results will be needed in the sequel.

### **Definition 12**

(Czerwik 1998) Let *X* be a (nonempty) set and $s\ge 1$ be a given real number. A function $d:X\times X\rightarrow {\mathbb {R}}^{+}$ is a *b*-metric iff, for all $x,y,z\in X$, the following conditions are satisfied:

$$(b_1)$$
$$d(x,y)=0$$ iff $$x=y,$$
$$(b_2)$$
$$d(x,y)=d(y,x),$$
$$(b_3)$$
$$d(x,z)\le s[d(x,y)+d(y,z)].$$

The pair (*X*, *d*) is called a *b*-metric space.

Note that, the class of *b*-metric spaces is effectively larger than the class of metric spaces, since a *b*-metric is a metric, when $s=1.$

The following example shows that in general a *b*-metric need not necessarily be a metric (see, also, Singh and Prasad 2008, p. 264).

### *Example 13*

(Aghajani et al. 2014) Let (*X*, *d*) be a metric space, and $\rho (x,y)=(d(x,y))^{p},$ where $p>1$ is a real number. Then, $\rho$ is a *b* -metric with $s=2^{p-1}.$

However, if (*X*, *d*) is a metric space, then $(X,\rho )$ is not necessarily a metric space.

For example, if $X= {\mathbb {R}}$ is the set of real numbers and $d(x,y)=\left| x-y\right|$ is the usual Euclidean metric, then $\rho (x,y)=(x-y)^{2}$ is a *b*-metric on ${\mathbb {R}}$ with $s=2,$ but not a metric on ${\mathbb {R}}$.

### **Definition 14**

Let *X* be a nonempty set. Then $({X},d,\preceq )$ is called a partially ordered *b*-metric space if and only if *d* is a *b*-metric on a partially ordered set $(X,\preceq ).$

### **Definition 15**

(Boriceanu et al. 2010) Let (*X*, *d*) be a *b*-metric space. Then a sequence $\{x_{n}\}$ in *X* is called *b*-convergent if and only if there exists $x\in X$ such that $d(x_n,x)\rightarrow 0$, as $n\rightarrow +\infty$. In this case, we write $\lim \nolimits _{n\rightarrow \infty }x_n=x.$

### **Definition 16**

(Boriceanu et al. 2010) Let (*X*, *d*) be a *b*-metric space. Then a sequence $\{x_{n}\}$ in *X* is called *b*-Cauchy if and only if $d(x_n,x_m)\rightarrow 0,$ as $n,m\rightarrow +\infty.$

### **Proposition 17**

(See, Remark 2.1 in Boriceanu et al. 2010) *In ab*-*metric space* (*X*, *d*) *the following assertions hold:*

(i): *Ab*-*convergent sequence has a unique limit.*
(ii): *Eachb*-*convergent sequence isb*-*Cauchy.*
(iii): *In general, ab*-*metric need not be continuous.*

### **Definition 18**

(Boriceanu et al. 2010) The *b*-metric space (*X*, *d*) is *b*-complete if every *b* -Cauchy sequence in *Xb*-converges.

### **Definition 19**

Let (*X*, *d*) and $(X^{\prime },d^{\prime })$ be two b-metric spaces. Then a function $f:X\rightarrow X^{\prime }$ is *b*-continuous at a point $x\in X$ if and only if it is *b*-sequentially continuous at *x*, that is, whenever $\{x_n\}$ is *b*-convergent to *x*, $\{f(x_n)\}$ is *b*-convergent to *f*(*x*).

### **Definition 20**

The function $\varphi :[0,\infty )\rightarrow [0,\infty )$ is called an Ultra-altering distance function, If the following conditions hold.

1. $$\varphi$$ is continuous
2. $$\varphi (0)\ge 0$$, and $$\varphi (t)\ne 0,t\ne 0$$.

In 2014 Ansari (2014) introduced the concept of *C*-class functions which cover a large class of contractive conditions.

### **Definition 21**

(Ansari 2014) A mapping $F:[0,\infty )^{2}\rightarrow {\mathbb {R}}$ is called a *C*-*class* function if it is continuous and satisfies following axioms:

1. $$F(r,t)\le r$$;
2. $F(r,t)=r$ implies that either $r=0$ or $t=0$; for all $r,t\in [0,\infty )$.

We denote a *C*-class functions as ${\mathcal {C}}$.

### *Example 22*

(Ansari 2014) The following functions $F:[0,\infty )^{2}\rightarrow {\mathbb {R}}$ are elements of ${\mathcal {C}}$, for all $r,t\in [0,\infty )$:

1. $$F(r,t)=mr$$, $$0{<}m{<}1$$, $$F(r,t)=r\Rightarrow r=0$$;
2. $$F(r,t)=r-t$$, $$F(r,t)=r\Rightarrow t=0$$;
3. $$F(r,t)=\frac{r}{(1+t)^{\alpha }}$$; $$\alpha \in (0,\infty )$$, $$F(r,t)=r$$$$\Rightarrow$$$$r=0$$ or $$t=0$$.

### **Lemma 23**

(Jovanović et al. 2010, Lemma 3.1) *Let*$\{x_{n}\}$*be a sequence in a metric type space* (*X*, *D*, *s*) *such that*

$$\begin{aligned} D(x_{n},x_{n+1})\le \beta D(x_{n-1},x_{n}) \end{aligned}$$

*for some*$\beta ,0<\beta <\frac{1}{s}$, *and each*$n=1,2,\cdots$. *Then*$\{x_{n}\}$*is a Cauchy sequence in* (*X*, *D*, *s*).

Motivated by the works in Nashine and Samet (2011), Shatanawi and Samet (2011) and Jamal (2015), In this paper, by using the *C*-class functions and a new approach, we present some coincidence point results for four mappings satisfying generalized $(\psi ,\phi )$-weakly contractive condition in the setting of ordered b-metric spaces where $\psi$ is altering distance function and $\varphi$ is Ultra-altering distance function. Also, an application and example are given to support our results.

## Main results

Let $(X,\preceq ,d)$ be an ordered b-metric space and $f, g, T, h:X\rightarrow X$ be four self mappings. In this paper, let

1 $$\begin{aligned} {\mathcal {N}}(x,y)\in \{d(hx,Ty),d(hx,fx),d(Ty,gy),d(hx,gy),d(Ty,fx)\} \end{aligned}$$

for all $$x,y\in X$$.

### **Theorem 24**

*Let*$(X,\preceq ,d)$*be an ordered completeb*-*metric space (with parametr*$s>1$*). Let*$f, g, T,h:X\rightarrow X$*be four mappings such that*$f(X)\subseteq T(X)$*and*$g(X)\subseteq h(X)$. *Suppose that for every*$x,y\in X$*with comparable elementshx*, *Ty*, *there exists*${\mathcal {N}}(x,y)$*such that*

2 $$\begin{aligned} \psi \big (s^{a}d(fx,gy)\big )\le F\big (\psi \big ({\mathcal {N}}(x,y)\big ),\varphi \big ({\mathcal {N}}(x,y)\big )\big ), \end{aligned}$$

*where*$\psi$*is altering distance function and*$\varphi$*is Ultra altering distance function*, $a > 1$*andFis aC*-*class function such thatFis increasing with respect to first variable and decreasing with respect to second variable. Letf*, *g*, *Tandhare continuous, the pairs* (*f*, *h*) *and* (*g*, *T*) *are compatible and the pairs* (*f*, *g*) *and* (*g*, *f*) *are partially weakly increasing with respect toTandh* , *respectively. Then, the pairs* (*f*, *h*) *and* (*g*, *T*) *have a coincidence pointwinX*. *Moreover, ifRwandSware comparable, thenwis a coincidence point off*, *g*, *Tandh*.

### *Proof*

Let $x_{0} \in X$ be an arbitrary point. Since $f(X)\subseteq T(X)$ and $g(X)\subseteq h(X)$, one can find $x_{1}, x_{2} \in X$ such that $fx_{0}= Tx_{1}$ and $gx_{1}=hx_{2}$.

Continuing this process, we construct a sequence $\{w_{n}\}$ defined by:

$$\begin{aligned} w_{2n+1}=Tx_{2n+1}=fx_{2n} \end{aligned}$$

and

$$\begin{aligned} w_{2n+2}=hx_{2n+2}=gx_{2n+1} \end{aligned}$$

for all $$n\ge 0$$.

Since, $x_{1}\in T^{-1}(fx_{0})$ and $x_{2}\in h^{-1}(gx_{1})$, and the pairs (*g*, *f*) and (*f*, *g*) are partially weakly increasing with respect to *T* and *h*, respectively, we have,

$$\begin{aligned} Tx_{1}=fx_{0}\preceq gx_{1}=hx_{2}\preceq fx_{2}=Tx_{3}. \end{aligned}$$

Repeating this process, we obtain $w_{2n+1}\preceq w_{2n+2}$ for all $n\ge 0.$

The proof will be done in three steps.

*Step I* We will show that $\lim \nolimits _{k\rightarrow \infty }d(w_{k},w_{k+1})=0.$

Define $d_{k}=d(w_{k},w_{k+1})$. Suppose $d_{k_{0}}=0$ for some $k_{0}$. Then, $w_{k_{0}}=w_{k_{0}+1}$. In case that $k_{0}=2n,$ then $w_{2n}=w_{2n+1}$ which gives $w_{2n+1}=w_{2n+2}$. Indeed,

3 $$\begin{aligned} \psi \big (s^{a}d(w_{2n+1},w_{2n+2})\big )&= \psi \big (s^{a}d(fx_{2n},gx_{2n+1})\big )\nonumber \\& \le F(\psi \big ({\mathcal {N}}(x_{2n},x_{2n+1})\big ),\varphi \big ({\mathcal {N}}(x_{2n},x_{2n+1}) \big )), \end{aligned}$$

where,

$$\begin{aligned} {\mathcal {N}}(x_{2n},x_{2n+1}) &\in \left\{ \begin{array}{l} d(hx_{2n},Tx_{2n+1}),d(hx_{2n},fx_{2n}),d(Tx_{2n+1},gx_{2n+1}), \\ d(hx_{2n},gx_{2n+1}),d(Tx_{2n+1},fx_{2n})\\ \end{array} \right\} \\&=\left\{ \begin{array}{l} d(w_{2n},w_{2n+1}),d(w_{2n},w_{2n+1}),d(w_{2n+1},w_{2n+2})\\ d(w_{2n},w_{2n+2}),d(w_{2n+1},w_{2n+1})\\ \end{array} \right\} \\&= \{0,d(w_{2n+1},w_{2n+2}),d(w_{2n},w_{2n+2})\} \end{aligned}$$

Taking ${\mathcal {N}}(x_{2n},x_{2n+1})=d(w_{2n+1},w_{2n+2}),$ then from (3) we have,

4 $$\begin{aligned} \psi \big (s^{a}d(w_{2n+1},w_{2n+2})\big )& \le F(\psi \big (d(w_{2n+1},w_{2n+2})\big),\varphi \big (d(w_{2n+1},w_{2n+2})\big )) \nonumber \\&\le \psi \big (d(w_{2n+1},w_{2n+2})\big ) \end{aligned}$$

which implies that $\psi (d(w_{2n+1},w_{2n+2}))=0$ or $\varphi (d(w_{2n+1},w_{2n+2}))=0$, that is, $w_{2n}=w_{2n+1}=w_{2n+2}$. Similarly, if $k_{0}=2n+1,$ then $w_{2n+1}=w_{2n+2}$ gives $w_{2n+2}=w_{2n+3}$. Consequently, the sequence $\{w_{k}\}$ becomes constant for $k\ge k_{0}$ and $w_{k_{0}}$ is a coincidence point of the pairs (*f*, *h*) and (*g*, *T*). For this aim, let $k_{0}=2n$. Since, $w_{2n}=w_{2n+1}=w_{2n+2},$ therefore,

$$\begin{aligned} w_{2n}=hx_{2n}=w_{2n+1}=Tx_{2n+1}=fx_{2n}=w_{2n+2}=gx_{2n+1}=hx_{2n+2}. \end{aligned}$$

This means that, $h(x_{2n})=f(x_{2n})$ and $T(x_{2n+1})=g(x_{2n+1}).$

On the other hand, the pairs (*f*, *h*) and (*g*, *T*) are compatible. So, they are weakly compatible. Hence, $fh(x_{2n})=hf(x_{2n})$ and $gT(x_{2n+1})=Tg(x_{2n+1})$, or, equivalently, $fw_{2n}=hw_{2n+1}$ and $gw_{2n+1}=Tw_{2n+2}$. Now, since, $w_{2n} =w_{2n+1} =w_{2n+2},$ we have, $fw_{2n}=hw_{2n}$ and $gw_{2n}=Tw_{2n}$.

In the other case, when $k_{0}=2n+1$, similarly, one can show that $w_{2n+1}$ is a coincidence point of the pairs (*f*, *h*) and (*g*, *T*). Also for ${\mathcal {N}}(x_{2n},x_{2n+1})=0$ or ${\mathcal {N}}(x_{2n},x_{2n+1})=d(w_{2n},w_{2n+2})$, one can obtain the desired result.

Now, suppose that

5 $$\begin{aligned} d_{k}=d(w_{k},w_{k+1})>0 \end{aligned}$$

for each *k*. Then we claim that

6 $$\begin{aligned} d(w_{k+1},w_{k+2})\le d(w_{k},w_{k+1}) \end{aligned}$$

for each $$k=1,2,3,\ldots.$$

To prove the claim, let $k=2n$, for an $n\ge 0$, assume that $d(w_{2n+1},w_{2n+2})\ge d(w_{2n},w_{2n+1})>0$. Then, as $hx_{2n}\preceq Tx_{2n+1}$, using (2) we obtain that,

7 $$\begin{aligned} \psi \big (s^{a}d(w_{2n+1},w_{2n+2})\big )&= \psi \big (s^{a}d(fx_{2n},gx_{2n+1})\big ) \nonumber \\& \le F(\psi \big ({\mathcal {N}}(x_{2n},x_{2n+1})\big ),\varphi \big ({\mathcal {N}}(x_{2n},x_{2n+1}) \big )), \end{aligned}$$

where,

$$\begin{aligned} {\mathcal {N}}(x_{2n},x_{2n+1}) &\in \left\{ \begin{array}{l} d(hx_{2n},Tx_{2n+1}),d(hx_{2n},fx_{2n}),d(Tx_{2n+1},gx_{2n+1}),\\ d(hx_{2n},gx_{2n+1}),d(Tx_{2n+1},fx_{2n})\\ \end{array} \right\} \\&=\{d(w_{2n},w_{2n+1}),d(w_{2n+1},w_{2n+2}),d(w_{2n},w_{2n+2}),0\}. \end{aligned}$$

If

$$\begin{aligned} {\mathcal {N}}(x_{2n},x_{2n+1})=d(w_{2n+1},w_{2n+2}), \end{aligned}$$

Then from (7), we have,

8 $$\begin{aligned} \psi \big (s^{a}d(w_{2n+1},w_{2n+2})\big ) \le F(\psi \big (d(w_{2n+1},w_{2n+2})\big ),\varphi \big (d(w_{2n+1},w_{2n+2})\big )) \end{aligned}$$

From definition of *F*, $\psi$ we get that

$$\begin{aligned} \psi \big (d(w_{2n+1},w_{2n+2})\big )&\le \psi \big (s^{a}d(w_{2n+1},w_{2n+2})\big ) \nonumber \\&\le F(\psi \big (d(w_{2n+1},w_{2n+2})\big ),\varphi \big (d(w_{2n+1},w_{2n+2})\big )\nonumber \\&\le \psi \big (d(w_{2n+1},w_{2n+2})\big ) \end{aligned}$$

Hence, $$F(\psi \big (d(w_{2n+1},w_{2n+2})\big ),\varphi \big ( d(w_{2n+1},w_{2n+2})\big )=\psi \big (d(w_{2n+1},w_{2n+2})\big )$$

which implies that,

$$\begin{aligned} \varphi \big (d(w_{2n+1},w_{2n+2})\big )=0, \end{aligned}$$

or

$$\begin{aligned} \psi \big (d(w_{2n+1},w_{2n+2})\big )=0, \end{aligned}$$

that is $d(w_{2n+1},w_{2n+2})=0$ a contradiction to (5). Hence,

$$\begin{aligned} d(w_{2n+1},w_{2n+2})\le d(w_{2n},w_{2n+1}), \text{ for } \text{ all } n\ge 0. \end{aligned}$$

Thus, (6) is proved for $k=2n$.

Using argument similar to the above, one can show the inequality (6) is true for $k=2n+1$. Therefore, (6) is true for all $k=1,2,3,\cdots$.

From definition of *F*, and condition (2) we get that

9 $$\begin{aligned} \psi \big (s^{a}d(w_{k+1},w_{k+2})\big )& \le F(\psi \big (d(w_{k+1},w_{k+2}) \big ),\varphi \big (d(w_{k+1},w_{k+2})\big )\nonumber \\&\le \psi \big (d(w_{k+1},w_{k+2})\big ) \end{aligned}$$

Thus, from the monotonocity increasing of $\psi$ we have for all $k\ge 0$

10 $$\begin{aligned} d(w_{k+1},w_{k+2})\le \frac{1}{s^{a}}d(w_{k},w_{k+1}) \end{aligned}$$

Analogously, in all cases, we see that $\{d(w_{k},w_{k+1})\}$ is a non-increasing sequence of nonnegative real numbers. Therefore, there is an $r\ge 0$ such that

11 $$\begin{aligned} \lim \limits _{k\rightarrow \infty }d(w_{k},w_{k+1})=r. \end{aligned}$$

We know that,

$$\begin{aligned}{\mathcal {N}}(x_{2n},x_{2n+1}) &\in \left\{ \begin{array}{l} d(hx_{2n},Tx_{2n+1}),d(hx_{2n},fx_{2n}),d(Tx_{2n+1},gx_{2n+1}),\\ d(hx_{2n},gx_{2n+1}),d(Tx_{2n+1},fx_{2n}) \end{array} \right\} \\& = \left\{ \begin{array}{l} d(w_{2n},w_{2n+1}),d(w_{2n},w_{2n+1}),d(w_{2n+1},w_{2n+2}),\\ d(w_{2n},w_{2n+2}),d(w_{2n+1},w_{2n+1})\\ \end{array} \right\}. \end{aligned}$$

Taking the limit as ${n\rightarrow \infty }$ in above and (9), we have

$$\begin{aligned} \psi \big (r\big ) \le F(\psi \big (r\big ),\varphi \big (r\big )), \end{aligned}$$

which implies that,

$$\begin{aligned} \psi \big (r\big )=0, \end{aligned}$$

that is $r=0$, therefore

12 $$\begin{aligned} r=\lim \limits _{k\rightarrow \infty }d(w_{k},w_{k+1})=\lim \limits _{n\rightarrow \infty }d(w_{2n},w_{2n+1})=0. \end{aligned}$$

*Step II* Using 10 and Lemma (23) we get $\{w_{n}\}$ is a *b*-Cauchy sequence in *X*.

*Step III* In this step we prove that *f*, *g*, *T* and *h* have a coincidence point.

Since $\{w_{n}\}$ is a *b*-Cauchy sequence in the complete *b*-metric space *X*, there exists $w\in X$ such that

13 $$\begin{aligned} \lim \limits _{n\rightarrow \infty }d(w_{2n+1},w)=\lim \limits _{n\rightarrow \infty }d(Tx_{2n+1},w)=\lim \limits _{n\rightarrow \infty }d(fx_{2n},w)=0 \end{aligned}$$

and

14 $$\begin{aligned} \lim \limits _{n\rightarrow \infty }d(w_{2n+2},w)=\lim \limits _{n\rightarrow \infty }d(hx_{2n+2},w)=\lim \limits _{n\rightarrow \infty }d(gx_{2n+1},w)=0. \end{aligned}$$

Hence,

15 $$\begin{aligned} hx_{2n}\rightarrow w~~\hbox {and}~fx_{2n}\rightarrow w, \quad \hbox {as } n\rightarrow \infty. \end{aligned}$$

As (*f*, *h*) is compatible, so,

16 $$\begin{aligned} \lim \limits _{n\rightarrow \infty }d(hfx_{2n},fhx_{2n})=0. \end{aligned}$$

Moreover, from $$\lim \limits _{n\rightarrow \infty }d(fx_{2n},w)=0,$$$$\lim \limits _{n\rightarrow \infty }d(hx_{2n},w)=0$$ and the continuity of *h* and *f*, we obtain,

17 $$\begin{aligned} \lim \limits _{n\rightarrow \infty }d(hfx_{2n},hw)=0=\lim \limits _{n\rightarrow \infty }d(fhx_{2n},fw). \end{aligned}$$

By the triangle inequality, we have,

18 $$\begin{aligned} d(hw,fw)& \le s[d(hw,hfx_{2n})+d(hfx_{2n},fw)] \nonumber \\&\le sd(hw,hfx_{2n})+s^2[d(hfx_{2n},fhx_{2n})+d(fhx_{2n},fw)]. \end{aligned}$$

Taking the limit as $n\rightarrow \infty$ in (18), we obtain that

$$\begin{aligned} d(hw,fw)\le 0, \end{aligned}$$

which yields that $fw=hw$, that is *w* is a coincidence point of *f* and *h*.

Similarly, it can be proved that $gw=Tw$. Now, let *Tw* and *hw* are comparable. By (2) we have,

19 $$\begin{aligned} \psi \big (s^{a}d(fw,gw)\big )\le F(\psi \big ({\mathcal {N}}(w,w)\big ),\varphi \big ({\mathcal {N}}(w,w) \big ), \end{aligned}$$

where,

$$\begin{aligned} {\mathcal {N}}(w,w) &\in \{d(hw,Tw),d(hw,fw),d(Tw,gw),d(hw,gw),d( Tw,fw)\} \\& =\{d(fw,gw),0\}. \end{aligned}$$

if

$$\begin{aligned} {\mathcal {N}}(w,w)=d(fw,gw) \end{aligned}$$

so (19) yields that

$$\begin{aligned} \psi \big (d(fw,gw)\big )& \le \psi \big (s^{a}d(fw,gw)\big )\\&\le F\left(\psi \big (d(fw,gw)\big ),\varphi \big (d(fw,gw)\big )\right)\\& \le \psi \big (d(fw,gw)\big ) \end{aligned}$$

which implies $\left[F(\psi \left( {d(fw,gw)} \right),\varphi \left( {d(fw,gw)) = \psi (d(fw,gw)} \right)\right]$, hence, either

$\psi \big (d(fw,gw)\big ) = 0\hbox { or }\varphi \big (d(fw,gw)\big )=0$, then in both cases we get $fw=gw$.

If ${\mathcal {N}}(w,w)=0$, then (19) yields that

$$\begin{aligned} \psi \left (s^{a}d(fw,gw)\right ) \le F(\psi \left (0\right ),\varphi \left (0\right ))\le F(0,\varphi \left (0\right ))\le 0 \end{aligned}$$

which implies $\psi \big (s^{a}d(fw,gw)\big ) =0$, and so $fw=gw$. So, in all cases we get that, $fw=gw=hw=Tw$. $\square$

By taking $\psi (t)=\varphi (t)=t$ and $F(r,t)=\lambda r$, $\lambda > 1,$ we get the following corollary.

### **Corollary 25**

*Let*$(X,\preceq ,d)$*be an ordered completeb*-*metric space (with parametr*$s>1$*). Let*$f,g,T,h:X\rightarrow X$*be four mappings such that*$f(X)\subseteq T(X)$*and*$g(X)\subseteq h(X)$. *Suppose that for every*$x,y\in X$*with comparable elementshx*, *Ty*, *there exists*${\mathcal {N}}(x,y)$*such that*

$$\begin{aligned} d(fx,gy)\le \frac{\lambda }{s^{a}}{\mathcal {N}}(x,y) \end{aligned}$$

*where*$\ a>1$*and*$\lambda > 1$. *Letf*, *g*, *Tandhare continuous, the pairs* (*f*, *h*) *and* (*g*, *T*) *are compatible and the pairs* (*f*, *g*) *and* (*g*, *f*) *are partially weakly increasing with respect toTandh*, *respectively. Then, the pairs* (*f*, *h*) *and* (*g*, *T*) *have a coincidence pointzinX*. *Moreover, ifTwandhware comparable, thenwis a coincidence point off*, *g*, *Tandh*.

In the following theorem, we replace the compatibility of the pairs (*f*, *h*) and (*g*, *T*) by weak compatibility of the pairs and we omit the continuity assumption of *f*, *g*, *T* and *h* and

### **Theorem 26**

*Let*$(X,\preceq ,d)$*be a regular partially orderedb*-*metric space (with parametr*$s>1$*)*, $f,g,T,h:X\rightarrow X$*be four mappings such that*$f(X)\subseteq T(X)$ and $g(X)\subseteq h(X)$*andTXandhXare complete subsets ofX*. *Suppose that for comparable elements*$hx,Ty\in X$, *we have*,

20 $$\begin{aligned} \psi \big (s^{a}d(fx,gy)\big )\le F(\psi \big ({\mathcal {N}}(x,y)\big ),\varphi \big ({\mathcal {N}}(x,y) \big )), \end{aligned}$$

*where*$\psi$*is altering distance function and*$\varphi$*is Ultra altering distance function and*$a{>}1$*andFisC*-*class function such thatFis increasing with respect to first varaible. Then, the pairs* (*f*, *h*) *and* (*g*, *T*) *have a coincidence pointwinXprovided that the pairs* (*f*, *h*) *and* (*g*, *T*) *are weakly compatible and the pairs* (*f*, *g*) *and* (*g*, *f*) *are partially weakly increasing with respect toTandh*, *respectively. Moreover, ifTwandhware comparable, then*$w\in X$*is a coincidence point off*, *g*, *Tandh*.

### *Proof*

Following to the construction of the sequence $w_{n}$ in the proof of Theorem (24), there exists $w\in X$ such that

21 $$\begin{aligned} \lim \limits _{k\rightarrow \infty }d(w_{k},w)=0. \end{aligned}$$

Since *T*(*X*) is complete and $\{w_{2n+1}\}\subseteq T(X)$, this implies that $w\in T(X)$. Hence, there exists $u\in X$ such that $w=Tu$ and

22 $$\begin{aligned} \lim \limits _{n\rightarrow \infty }d(w_{2n+1},Tu)=\lim \limits _{n\rightarrow \infty }d(Tx_{2n+1},Tu)=0. \end{aligned}$$

Similarly, there exists $v\in X$ such that $w=Tu=hv$ and

23 $$\begin{aligned} \lim \limits _{n\rightarrow \infty }d(w_{2n},hv)=\lim \limits _{n\rightarrow \infty }d(hx_{2n},hv)=0. \end{aligned}$$

We prove that *v* is a coincidence point of *f* and *h*.

Since $Tx_{2n+1}\rightarrow w=hv$, as ${n\rightarrow \infty }$ and the regularity of *X*, $Tx_{2n+1}\preceq hv$. But from triangle inequality of *b* -metric space we have $d(fv,w) \le sd(w,gx_{2n+1}) + sd(fv,gx_{2n+1})$

Therefore, from (20) and the monotonocity increasing of $\psi$ we have

24 $$\begin{aligned} \psi \big (d(fv,w)-sd(w,gx_{2n+1})\big )&\le \psi \big (sd(fv,gx_{2n+1})\big ) \le \psi \big (s^{a}d(fv,gx_{2n+1})\big ) \\&\le F(\psi \big ({\mathcal {N}}(v,x_{2n+1})\big ),\varphi \big ({\mathcal {N}}(v,x_{2n+1})\big )), \nonumber \end{aligned}$$

where, from 1,

$$\begin{aligned}&{\mathcal {N}}(v,x_{2n+1}) \in \{d(hv,Tx_{2n+1}),d(hv,fv),d(Tx_{2n+1},gx_{2n+1}),d(hv,gx_{2n+1}),d(Tx_{2n+1},fv)\}\\&\quad \rightarrow \{0,d(w,fv)\}. \end{aligned}$$

Taking the limit as ${n\rightarrow \infty }$ in (24), using 1 and the continuity of $\psi$ and $\varphi$, we get the following two case:

*Case(1)*

$$\begin{aligned} \psi (d(fv,w))\le F\big (\psi (d(w,fv)),\varphi (d(w,fv))\big ) \le \psi (d(w,fv)) \end{aligned}$$

so, $$fv=w=hv$$.

*Case(2)*

$$\begin{aligned} \psi \big (d(fv,w)\le F(\psi \big (0\big ),\varphi \big (0\big ))\le \psi \big (0 \big )=0 \end{aligned}$$

so, $$fv=w=hv$$.

As *f* and *h* are weakly compatible, we have $fw=fhv=hfv=hw.$ Thus, *w* is a coincidence point of *f* and *h*.

Similarly it can be shown that *w* is a coincidence point of the pair (*g*, *T*).

The rest of the proof can be done using similar arguments as in Theorem 24. $\square$

Taking $h=T$ in Theorem 24, we obtain the following result.

### **Corollary 27**

*Let*$(X,\preceq ,d)$*be a partially ordered completeb*-*metric space (with parametr*$s>1$*)and*$f,g,T:X\rightarrow X$*be three mappings such that*$f(X)\cup g(X)\subseteq T(X)$*andTis continuous. Suppose that for every*$x,y\in X$*with comparable elementsTx*, *Ty*, *we have*,

25 $$\begin{aligned} \psi \big (s^{a}d(fx,gy)\big )\le F(\psi \big ({\mathcal {N}}(x,y)\big ),\varphi \big ({\mathcal {N}}(x,y) \big )), \end{aligned}$$

*where*,

$$\begin{aligned} {\mathcal {N}}(x,y)\in \{d(Tx,Ty),d(Tx,fx),d(Ty,gy),d(Tx,gy),d(Ty,fx)\} \end{aligned}$$

*where*$\psi$*is altering distance function*, $\varphi$*is Ultra altering distance function*, ${a>}1$*andFisC*-*classfunction such thatFis increasing with respect to first variable.Then*, *f*, *gandThave a coincidence point inXprovided that the pair* (*f*, *g*) *is weakly increasing with respect toTand either*,

a.: *the pair* (*f*, *T*) *is compatible andfis continuous, or*,
b.: *the pair* (*g*, *T*) *is compatible andgis continuous*.

Taking $T=h$ and $f=g$ in Theorem 24, we obtain the following coincidence point result.

### **Corollary 28**

*Let*$(X,\preceq ,d)$*be a partially ordered completeb*-*metric space (with parameter*$s>1$*) and*$f,T:X\rightarrow X$*be two mappings such that*$f(X)\subseteq R(X)$. *Suppose that for every*$x,y\in X$*for whichTx*, *Tyare comparable, we have*,

26 $$\begin{aligned} \psi \left (s^{a}d(fx,fy)\right )\le F(\psi \left ({\mathcal {N}}(x,y)\right ),\varphi \left ({\mathcal {N}}(x,y) \right )), \end{aligned}$$

*where,*

$$\begin{aligned} {\mathcal {N}}(x,y)\in \{d(Tx,Ty),d(Tx,fx),d(Ty,fy),d(Tx,fy),d(Ty,fx)\}, \end{aligned}$$

$\psi$*is altering distance function*, $\varphi$*is Ultra altering distance function*, ${a>}1$*andFa isC*-*class function such thatFis increasing with respect to first variable. Then, the pair* (*f*, *T*) *has a coincidence point inXprovided thatfandTare continuous, the pair* (*f*, *T*) *is compatible andfis weakly increasing with respect toT*.

### *Example 29*

Let $F(r,t)=\frac{r}{1+t}$, $X=[0,\infty )$ and *d* on *X* be given by $d(x,y)=\left| x-y\right| ^{2},$ for all $x,y\in X$. We define an ordering “$\preceq$” on *X* as follows:

$$\begin{aligned} x\preceq y\Longleftrightarrow y\le x,\quad \forall x,y\in X. \end{aligned}$$

Define self-maps *f*, *g*, *h* and *T* on *X* by

$$\begin{aligned} \begin{array}{ll} fx=\ln (1+x), &{} Tx=\exp (7x)-1, \\ gx=\ln \left(1+\dfrac{x}{3}\right), & hx=\exp (21x)-1. \end{array} \end{aligned}$$

To prove that (*f*, *g*) is partially weakly increasing with respect to *T*, let $x,y\in X$ be such that $y\in T^{-1}fx$, that is, $Ty=fx$. By the definition of *f* and *T*, we have $\ln 1+x=\exp (7y)-1$ and $y=\frac{\ln (1+\ln (1+x))}{7}$. ,

$$\begin{aligned} fx=\ln (1+x)\ge \ln \left(1+\frac{1}{21}\ln \left(1+\ln (1+x)\right)\right)=\ln \left(1+\frac{y}{3}\right)=gy. \end{aligned}$$

Therefore, $fx\preceq gy$. Hence (*f*, *g*) is partially weakly increasing with respect to *T*.

To prove that (*g*, *f*) is partially weakly increasing with respect to *h*, let $x,y\in X$ be such that $y\in h^{-1}gx$. This means that $hy=gx$. Hence, we have $\ln \left(1+\dfrac{x}{3}\right)=\exp (21y)-1$ and so, $y=\frac{\ln (1+\ln (1+ \frac{x}{3}))}{21}$. , so,

$$\begin{aligned} gx=\ln \left( 1+\dfrac{x}{3}\right) \ge \ln \left( 1+\frac{\ln \left( 1+\ln \left( 1+\frac{x}{3}\right) \right) }{21} \right) =\ln (y)=fy. \end{aligned}$$

Therefore, $$gx\preceq fy$$.

Furthermore, $fX=gX=hX=TX=[0,\infty )$ and the pairs (*f*, *h*) and (*g*, *T*) are compatible. Indeed, let $\{x_{n}\}$ is a sequence in *X* such that $\lim \nolimits _{n\rightarrow \infty }d(t,fx_{n})=\lim \nolimits _{n\rightarrow \infty }d(t,hx_{n})=0,$ for some $t\in X$. Therefore, we have,

$$\begin{aligned} \lim \limits _{n\rightarrow \infty }\left| \ln (1+x_{n})-t\right| =\lim \limits _{n\rightarrow \infty }\left| \exp (21x_{n})-1-t\right| =0. \end{aligned}$$

Continuity of $\ln x$ and $\exp (21x)-1$ on *X* implies that,

$$\begin{aligned} \lim \limits _{n\rightarrow \infty }\left| x_{n}-\exp (t)+1\right| =\lim \limits _{n\rightarrow \infty }\left| x_{n}-\frac{\ln t+1}{21} \right| =0, \end{aligned}$$

and the uniqueness of the limit gives that $\exp (t)+1=\frac{\ln t+1}{21}.$ But,

$$\begin{aligned} \exp (t)-1=\frac{\ln t+1}{21}\Longleftrightarrow t=0. \end{aligned}$$

So, we have $t=0.$ Since *f* and *h* are continuous, we have

$$\begin{aligned} \lim \limits _{n\rightarrow \infty }d(fhx_{n},hfx_{n})=\lim \limits _{n\rightarrow \infty }\left| fhx_{n}-hfx_{n}\right| ^{2}=0. \end{aligned}$$

Define $\psi ,\varphi :[0,\infty )\rightarrow [0,\infty )$ as $\psi (t)=\frac{441}{256}t$ and $\varphi (t)=\frac{313}{128}$ for all $t\in [0,\infty ).$

Using the mean value theorem for the functions $\ln (1+z)$ and $\exp (z)$ on the intervals $[x,\tfrac{y}{3}]\subset X$ and $[21x,7y]\subset X,$ respectively, we have,

$$\begin{aligned} \psi (2^{7} d(fx,gy))&= 2^{7}\frac{441}{256}\left| fx-gy\right| ^{2}=\frac{441}{ 2}\left| \ln (1+x) -\ln (1+\dfrac{y}{3})\right| ^{2} \nonumber \\&\le \frac{441}{2}\left| x-\frac{y}{3}\right| ^{2}\le \frac{441}{2 }\frac{\left| 21x-7y\right| ^{2}}{441} \\&\le \frac{1}{2}\left| \exp (21x)-1-\exp (7y)-1\right| ^{2}\le \frac{1}{2}\left| hx-Ty\right| ^{2} \\& = \frac{1}{2}d(hx,Ty)=\frac{\psi (d(hx,Ty))}{1+\varphi (d(hx,Ty)) } = F(\psi (d(hx,Ty)), \varphi (d(hx,Ty))). \end{aligned}$$

Thus, (2) is satisfied for all $x,y\in X$ with $a = 7$ and ${\mathcal {N}}(x,y)=d(hx,Ty).$ Therefore, all the conditions of Theorem 24 are satisfied. Moreover, 0 is a coincidence point of *f*, *g*, *T* and *h*. $\square$

### **Corollary 30**

*Let*$(X,\preceq ,d)$*be a regular partially orderedb*-*metric space (with parametr*$s>1$*)*, $f,g,T:X\rightarrow X$*be three mappings such that*$f(X)\subseteq T(X)$*and*$g(X)\subseteq T(X)$*andTXis a complete subset ofX*. *Suppose that for comparable elements*$Tx,Ty\in X$, *we have,*

27 $$\begin{aligned} \psi \left (s^{a}d(fx,gy)\right )\le F(\psi \left ({\mathcal {N}}(x,y)\right ),\varphi \left ({\mathcal {N}}(x,y) \right ), \end{aligned}$$

*where*

$$\begin{aligned} {\mathcal {N}}(x,y)\in \{d(Tx,Ty),d(Tx,fx),d(Ty,gy),d(Tx,gy),d(Ty,fx)\} \end{aligned}$$

*where*$\psi$*is altering distance function*, $\varphi$*is Ultra altering distance function*, ${a>}1$*andFisC*-*class function such thatFis increasing with respect to first variable. Then, the pairs* (*f*, *T*) *and* (*g*, *T*) *have a coincidence pointwinXprovided that the pair* (*f*, *g*) *is weakly increasing with respect toT*.

### **Corollary 31**

*Let*$(X,\preceq ,d)$*be a regular partially orderedb*-*metric space (with parameter*$s>1$*)*, $f,T:X\rightarrow X$*be two mappings such that*$f(X)\subseteq T(X)$*andTXis a complete subset ofX*. *Suppose that for comparable elements*$Tx,Ty\in X$, *we have,*

28 $$\begin{aligned} \psi \big (s^{a}d(fx,fy)\big )\le F(\psi \big ( {\mathcal {N}}(x,y)\big ),\varphi \big ({\mathcal {N}}(x,y)\big )), \end{aligned}$$

*where,*

$$\begin{aligned} {\mathcal {M}}(x,y)\in \{d(Tx,Ty),d(Tx,fx),d(Ty,fy),d(Tx,fy),d(Ty,fx)\} \end{aligned}$$

$\psi$*is altering distance function*, $\varphi$*is Ultra altering distance function*, ${a>}1$*andFisC*-*class function such thatFis increasing with respect to first variable. Then, the pair* (*f*, *T*) *have a coincidence pointwinXprovided thatfis weakly increasing with respect toT*.

Taking $T=h=I_X$ (the identity mapping on *X*) in Theorems 24 and 26, we obtain the following common fixed point result.

### **Corollary 32**

*Let*$(X,\preceq ,d)$*be a partially ordered completeb*-*metric space(with parametr*$s>1$*). Let*$f,g:X\rightarrow X$*be two mappings. Suppose that for every comparable elements*$x,y\in X$,

29 $$\begin{aligned} \psi \big (s^{a}d(fx,gy)\big )\le F(\psi \big ( {\mathcal {N}}(x,y)\big ),\varphi \big ({\mathcal {N}}(x,y)\big )), \end{aligned}$$

*where,*

$$\begin{aligned} {\mathcal {N}}(x,y)\in \{d(x,y),d(x,fx),d(y,gy),d(x,gy),d(y,fx)\}, \end{aligned}$$

$\psi$*is altering distance function*, $\varphi$*is Ultra altering distance function*, ${a>}1$*andFisC*-*class function such thatFis increasing with respect to first variable. Then, the pair* (*f*, *g*) *have a common fixed pointwinXprovided that the pair* (*f*, *g*) *is weakly increasing and either*,

a.: *forgis continuous, or,*
b.: *Xis regular*.

## Application

In this section, we will use Corollary 32 to show that there is a solution to the following integral equations:

30 $$\begin{aligned} x(t)&= \int _{a}^{b} G(t,r)H_{1}(r,x(r)) dr; \,\,\,\, t\in [a,b] \nonumber \\ x(t)&= \int _{a}^{b} G(t,r)H_{2}(r,x(r)) dr; \,\,\,\, t\in [a,b] \end{aligned}$$

Let $X = (C[a,b],{\mathbf {R}})$ denote the set of all continuous functions from [*a*, *b*] to ${\mathbf {R}}$. Consider the partial order on *X* to be define as: $x, y \in X, \,\,\,\, x \preceq y \text{ iff } x(t) \le y(t), \,\,\, \forall t \in [a,b]$.

Define mappings $f,g: X \rightarrow X$ by

31 $$\begin{aligned} fx(t)= & {} \int _{a}^{b} G(t,r)H_{1}(r,x(r)) dr; \,\,\,\, t\in [a,b]\end{aligned}$$

32 $$\begin{aligned} gx(t)= & {} \int _{a}^{b} G(t,r)H_{2}(r,x(r)) dr; \,\,\,\, t\in [a,b] \end{aligned}$$

### **Theorem 33**

*Consider Equ. * (30) *and suppose:*

1. $G:[a,b]\times [a,b] \rightarrow [0,\infty )$*is a continuous function,*
2. $H_{1}, H_{2}:[a,b]\times {\mathbf {R}} \rightarrow {\mathbf {R}}$*are continuous functions,*
3. $$\sup _{t\in [a,b]}\int _{a}^{b}G(t,r)dr<\frac{1}{\sqrt{2^{m}}} ,m>1$$
4. *for all*$r \in [a,b]$*and*$x \in X$*we have*
  $$\begin{aligned} H_{1}(r,x(r))&\le H_{2}\Big (r,\int _{a}^{b}G(t,r)H_{1}(r,x(r)) dr\Big )\\ H_{2}(r,x(r))& \le H_{1}\Big (r,\int _{a}^{b}G(t,r)H_{2}(r,x(r)) dr\Big ) \end{aligned}$$
5. *For all*$x(r),y(r)\in X$*with*$x(r)\le y(r)$; $r\in [a,b]$*we have*
  $$\begin{aligned} |H_{1}(r,x(r))-H_{2}(r,y(r)|^{2}\le \sqrt{\ln (1+|x(r)-y(r)|^{2})}. \end{aligned}$$

*Then, the integral Eq. * (30) *have a solution*$x \in X$.

### *Proof*

Clearly from condition (4), the mappings *f*, *g* are weakly increasing with respect to $\preceq$. Let *X* and *f*, *g* be as defined above. For all $x, y \in X$ define the *b*-metric on *X* by

33 $$\begin{aligned} d(x,y) = \Big ( \sup _{t \in [a,b]} |x(t) - y(t)| \Big )^{2}. \end{aligned}$$

Clearly that (*X*, *d*) is a complete *b*-metric space with constant $( s=2)$. Moreover, in Nieto and Rodaiguez-Loez (2007) it is proved that $(X,\preceq )$ is regular.

Now let $x,y \in X$ such that $x\preceq y$, then from condition (5) above, for all $t \in [a,b]$ we have

$$\begin{aligned} d(fx,gy)&= \Big (\sup _{t\in [a,b]}|fx(t)-gy(t)|\Big )^{2} \\&= \Big (\sup _{t\in [a,b]}\big |\int _{a}^{b}G(t,r)|H_{1}(r,x(r))-H_{2}(r,y(r)|dr\big |\Big )^{2} \\&\le \Big (\sup _{t\in [a,b]}\int _{a}^{b}G(t,r)|H_{1}(r,x(r))-H_{2}(r,y(r)|dr\Big )^{2} \\& \le \Big (\sup _{t\in [a,b]}\int _{a}^{b}G(t,r)\sqrt{\ln (1+|x(r)-y(r)|^{2})}dr\Big )^{2} \\& \le \Big (\sup _{t\in [a,b]}\int _{a}^{b}G(t,r)\sqrt{\ln (1+d(x,y))}dr \Big )^{2} \\& \le \Big (\sup _{t\in [a,b]}\int _{a}^{b}G(t,r)dr\Big )^{2}\ln (1+d(x,y)) \\&\le \frac{1}{2^{m}}\ln (1+d(x,y)) \end{aligned}$$

Thus,

34 $$\begin{aligned} 2^{m}d(fx,gy)& \le \ln (1+d(x,y)) \nonumber \\&= d(x,y)-\big ( d(x,y)-\ln (1+d(x,y))\big) \end{aligned}$$

Now, by taking $\psi (t)=t$ and $\varphi (t)=t-\ln (1+t)$ and the function $F(r,t)=r-t$, then clearly $\psi$ is Altering distance function and $\varphi$ is Ultra- Altering distance function, also $F\in {\mathcal {C}}$. Therefore Eq. 34 becomes

$$\begin{aligned} \psi (s^{m}d(fx,gy))&= \psi (2^{m}d(fx,gy)) \\&= 2^{m}d(fx,gy) \\& \le d(x,y)-(d(x,y)-\ln (1+d(x,y))) \\&= \psi (d(x,y))-\varphi (d(x,y)) \\&= \psi ({\mathcal {N}}(x,y))-\varphi ({\mathcal {N}}(x,y)) \\&= F(\psi ({\mathcal {N}}(x,y)),\varphi ({\mathcal {N}}(x,y))) \end{aligned}$$

Therefore, all conditions of corollary 32 are satisfied with ${\mathcal {N}}(x,y) = d(x,y)$ and $a = m$. As a result of corollary 32 the mappings *f* and *g* has a common fixed point in *X* which is a solution of the Eq. 30. $\square$

## Conclusions

By using the *C*-class function *F* such that *F* is increasing with respect to first variable and decreasing with respect to second variable, we proved some coincidence point results for four continuous mappings *f*, *g*, *T* and *h*, where the pairs (*f*, *h*) and (*g*, *T*) are compatible satisfying generalized $(\psi ,\phi )$-weakly contractive condition in the setting of ordered *b*-metric spaces, $\psi$ is altering distance function and $\varphi$ is Ultra-altering distance function. Also, we can replace the compatibility of the pairs (*f*, *h*) and (*g*, *T*) by weak compatibility of the pairs and we omit the continuity assumption of *f*, *g*, *T* and *h*. This approach can be extended to other spaces.
